# Characterizing the Impact of Doppler Effects on Body-Centric LoRa Links with SDR

**DOI:** 10.3390/s21124049

**Published:** 2021-06-12

**Authors:** Thomas Ameloot, Marc Moeneclaey, Patrick Van Torre, Hendrik Rogier

**Affiliations:** 1 IDLab, Department of Information Technology (INTEC), Ghent University-imec, Technologiepark-Zwijnaarde 126, B-9052 Ghent, Belgium; patrick.vantorre@ugent.be (P.V.T.); hendrik.rogier@ugent.be (H.R.); 2 Department of Telecommunications and Information Processing (TELIN), Ghent University, Sint-Pietersnieuwstraat 41, B-9000 Ghent, Belgium; marc.moeneclaey@ugent.be

**Keywords:** Internet of Things, LoRa, body-centric communication, software defined radio, doppler

## Abstract

Long-range, low-power wireless technologies such as LoRa have been shown to exhibit excellent performance when applied in body-centric wireless applications. However, the robustness of LoRa technology to Doppler spread has recently been called into question by a number of researchers. This paper evaluates the impact of static and dynamic Doppler shifts on a simulated LoRa symbol detector and two types of simulated LoRa receivers. The results are interpreted specifically for body-centric applications and confirm that, in most application environments, pure Doppler effects are unlikely to severely disrupt wireless communication, confirming previous research, which stated that the link deteriorations observed in a number of practical LoRa measurement campaigns would mainly be caused by multipath fading effects. Yet, dynamic Doppler shifts, which occur as a result of the relative acceleration between communicating nodes, are also shown to contribute to link degradation. This is especially so for higher LoRa spreading factors and larger packet sizes.

## 1. Introduction

In recent years, sub-GHz low-power wide-area network (LPWAN) technologies such as SigFox [1], NB-IoT [2], and LoRa [3] have played key roles in the development of the rapidly evolving Internet of Things (IoT). Following the widespread adoption of these technologies in a wide range of application environments, several research efforts have been devoted to assessing the viability of using LoRa modulation for body-centric wireless communication [4,5,6]. LoRa employs wide-band frequency-modulated pulses called chirps to achieve spreading gain, which results in the successful reception of packets at extremely low signal-to-noise ratio (SNR) levels [7]. Compared to its competitors, LoRa is especially suitable for body-centric applications as its data rate can be adapted, which has been shown to benefit the coverage of body-centric LoRa networks [8]. LoRa modulation is most often deployed in the 868 MHz industrial, scientific and medical (ISM) radio band. For body-centric wireless communication, this band is very interesting as it both enables applications to benefit from the excellent propagation characteristics observed at sub-GHz frequencies, while still allowing compact wearable antennas to be designed, given the wavelength of 35 cm.

To properly assess the viability of using LoRa modulation in body-centric networks, it is important to thoroughly evaluate its physical layer performance. Unfortunately, as LoRa is a proprietary technology, research into its wireless performance has been challenging. Until now, it has mostly relied on either theoretical reviews [9,10,11,12] or channel measurements gathered by commercial transceivers [4,5,6,7]. Recently, efforts have been carried out to implement LoRa modulation in signal processing code, either for simulation purposes or for implementation on software-defined radio (SDR) platforms [13,14,15,16,17,18]. Having access to individual I and Q samples enables much more accurate channel estimation than is possible with commercial transceivers. Furthermore, using these implementations, different propagation mechanisms can be simulated, and their influence on LoRa modulation can be analyzed.

One mechanism that can impact body-centric wireless links is the Doppler effect. As people move around, their relative velocities change continuously. Several sources declare that LoRa shows good immunity against the Doppler effect [19,20]. Some practical studies confirm this, however, others do not. In [21], LoRa link degradations are demonstrated for relative velocities around 40 km/h. In [22], the Doppler effect is blamed for severe link degradations. In [23], the authors presented two body-to-base-station measurement campaigns at different velocities ( 6.2 km/h and 31.1 km/h), which show no significant difference. These contradictions were also examined in [24], which presents lab measurements and outdoor experiments investigating the Doppler effect in LoRa satellite communication. In [24], it is demonstrated that LoRa is indeed reasonably Doppler-resistant, and it is stated that the link degradations observed in [21] are expected to be the result of Doppler spread. However, most of these conclusions are based on fully experimental examinations.

This paper assesses the impact of Doppler effects on a recently developed SDR implementation of LoRa [25]. General conclusions are drawn based on computer simulations of static and dynamic Doppler effects, assuming a worst-case angle of incidence. For both cases, a comparison is made between two packet synchronization strategies. Results are also interpreted specifically for body-centric LoRa networks. Finally, guidelines are provided on how static and dynamic Doppler effects can be mitigated in LoRa networks, e.g., by modifying the packet structure. The paper is structured as follows. In Section 2, LoRa modulation is described in general. Additionally, key points on the SDR implementation applied in this work are presented. Section 3 elaborates on relevant considerations published in other research and describes the software-based simulation of Doppler effects on LoRa modulation. A discussion of the results in perspective of previous research is presented in Section 4. General conclusions are drawn in Section 5.

## 2. SDR-Based LoRa Modulation

### 2.1. LoRa Modulation Basics

LoRa is based on chirp spread spectrum (CSS) modulation [26], which uses wide-band frequency-modulated pulses to encode information. The most important modulation parameters are the spreading factor SF ∈{7,8,9,10,11,12}, bandwidth BW ∈{125,250,500}kHz, and code rate CR ∈{4/5,4/6,4/7,4/8}. In most LoRa research, the bandwidth is actually used to describe the frequency swing *B* of the signals, as very little energy is present for frequencies outside of the range described by *B*. The spreading factor determines the slope of the chirp w.r.t. the frequency swing. For a given *B*, a higher spreading factor spreads any given LoRa symbol over a longer symbol interval. A detailed description of LoRa packet air times and data rates for different SF values is provided in [8]. Increasing the spreading factor or the frequency swing also impacts the sensitivity of LoRa receivers, as presented in [27]. On the packet level, a default preamble length of 12.25 symbols is considered. This preamble consists of eight up-chirps that constitute the pilot sequence, two so-called sync word symbols, which enable the user to distinguish packets from different LoRa networks, and 2.25 down-chirps, which make up the start-of-frame delimiter (SFD), used for packet synchronization. The optional header and/or cyclic redundancy check that can be added to LoRa packets are considered to be part of the data payload. The interleaving and decoding steps that are applied to data when encapsulated in a LoRa packet have been documented in [13] and are considered to be part of the data link layer. Consequently, these are not discussed in this paper. Additional details and considerations on high-level aspects of LoRa and LoRaWAN can be found in [27,28].

### 2.2. Software Implementation

As mentioned earlier, this paper employs a software implementation of LoRa to investigate the impact of Doppler shifts on the physical layer of LoRa modulation. In this implementation, which is based on LoRa waveform expressions presented in [12,25], a discrete LoRa up-chirp with symbol energy $E_{s}$ and symbol duration $T_{s}$ is described by the waveform

(1) $$\begin{array}{cc} s_{\mathrm{up}}\left[n\right]=\sqrt{\frac{E_{s}}{N}}\; & \exp\;\left\{\;j\;2\pi \left[\frac{\alpha }{2}\frac{T_{s}^{2}n^{2}}{N^{2}}\right]\;\right\}, \end{array}$$

where n∈{0,1,2,...,N−1} signifies the sample index and *N* equals the amount of samples used to describe the up-chirp. *N* is related to the spreading factor through N=K·M, with $M=2^{\mathrm{SF}}$ and $K\in Z_{+}$ the oversampling factor. The slope α (in Hz/s) of the up-chirp is related to the symbol duration and the frequency swing *B* through $\alpha =B/T_{s}$. A LoRa symbol a∈{0,1,2,...,M−1} is encoded by changing the starting frequency of the chirp to aB/M and resetting the instantaneous frequency of the chirp to zero when it reaches *B*. According to the slope of the chirp, this occurs at the time instant when n=(1−a/M)N. After the reset, the chirp frequency again increases linearly according to α. Thus, the encoded LoRa symbol s[n,a] is described by

(2) $$\begin{array}{cc} s\left[n,a\right]=\sqrt{\frac{E_{s}}{N}}\;\exp & \;\left\{\;j\;2\pi \left[\frac{\alpha }{2}\frac{T_{s}^{2}n^{2}}{N^{2}}+F_{a}\left[n,a\right]\frac{T_{s}n}{N}\right]\;\right\}, \end{array}$$

(3) $$\begin{array}{cc} \mathrm{where}\;F_{a}\left[n,a\right] & =\left\{\begin{array}{cc} \frac{aB}{M} & \;n<(1\;-\;\frac{a}{M})N\\ \frac{aB}{M}-B & \;n>(1\;-\;\frac{a}{M})N \end{array}\right.. \end{array}$$

When no oversampling is applied (K=1, $N/T_{s}=B$ and N=M), this symbol can be detected by first multiplying s[n,a] by the complex conjugate (as indicated by the asterisk) of the up-chirp $s_{\mathrm{up}}\left[n\right]$ and then applying the discrete Fourier transform (DFT), indicated by the operator F, yielding

(4) $$\begin{array}{c} X_{i}=F\left\{s\left[n,a\right]\;\cdot \;s_{\mathrm{up}}^{*}\left[n\right]\right\}. \end{array}$$

As the signal $s_{\mathrm{up}}^{*}$ corresponds to a chirp with a frequency that linearly decreases, it will be referred to as the down-chirp $s_{\mathrm{down}}$ from now on. The frequency bins $X_{i}$ resulting from Equation (4) describe the possible symbol levels that can be obtained when detecting the LoRa symbol. An estimate $\tilde{a}$ for the encoded symbol *a* is found by determining

(5) $$\tilde{a}=\arg_{i}\;\max\left(\;\left|X_{i}\right|\;\right).$$

**Figure 1:**
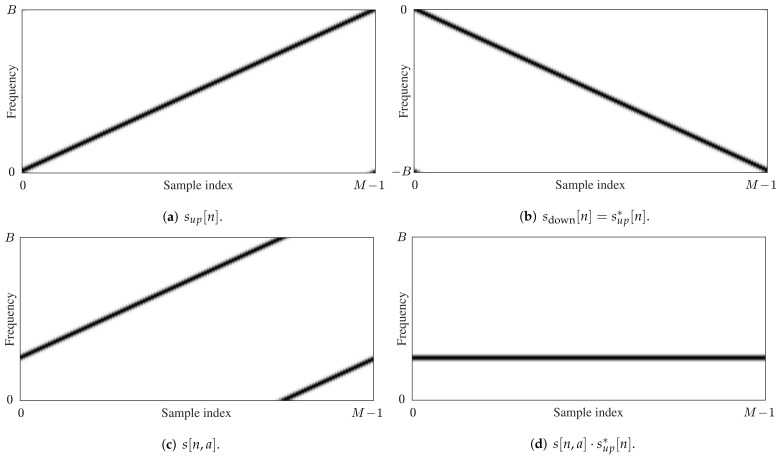
Spectrograms of the waveforms for an up-chirp $\;s_{\mathrm{up}}\left[n\right]$, a down-chirp $s_{\mathrm{down}}\left[n\right]=s_{\mathrm{up}}^{*}\left[n\right]$, an arbitrary LoRa symbol *s*[*n*,*a*], and the product $s[n,a]\cdot s_{\mathrm{up}}^{*}\left[n\right]$ as applied in (4), for *K* = 1 and *N* = *M*.

In Figure 1, spectrograms are shown for the chirps described by $s_{\mathrm{up}}\left[n\right]$, $s_{\mathrm{up}}^{*}\left[n\right]$ ($=s_{\mathrm{down}}\left[n\right]$), and the modulated symbol s[n,a], with *a* an arbitrary symbol value. Additionally, the result of the element-wise multiplication of s[n,a] and $s_{\mathrm{up}}^{*}\left[n\right]$ is shown, which illustrates how all symbol energy is concentrated in a single DFT bin when applying (4).

When oversampling is applied (K>1, $N/T_{s}=KB$, and N=KM), frequency shifts can be simulated without suffering from aliasing. However, as thoroughly described in [25], when detecting the symbol, the signal must first be filtered using an anti-aliasing filter and decimated such that N=M in order to apply the detection procedure presented above. Additionally, a frequency error Δf can be corrected by applying a frequency shift before filtering and decimating the signal. When $\Delta \tilde{f}$ denotes an estimate of Δf, this shift is applied by multiplying each sample by the following complex exponential:

(6) $$\exp\left\{-j2\pi \Delta \tilde{f}\frac{nT_{s}}{N}\right\}.$$

## 3. Doppler Effects Simulation and Analysis

In current literature on LoRa Doppler effects, contradictory conclusions are presented. The most thorough examinations of this topic are found in [21,24]. In [21], the impact of Doppler spread is first analyzed by comparing the coherence time of the channel to LoRa symbol durations for different modulation settings. Then, a LoRa receiver node is attached to a lathe, rotating it at high speeds to determine the impact of different angular velocities on the link quality. Finally, a long-range outdoor measurement campaign is presented. In [24], Doppler effects are analyzed in the context of satellite radio applications. Lab measurements are presented based on SDR-synthesized LoRa packets, received by an SX1276 LoRa transceiver. Additionally, an outdoor measurement campaign is performed as well. Whereas the authors of both papers consider the impact of Doppler effects on the full LoRa modulation protocol based on experimental data gathered with commercial transceiver modules, the present paper also focuses on the fundamental performance impact of the Doppler effects on the symbol detector itself. This standalone symbol detector is implemented in software and assumes perfect packet detection and synchronization. Additionally, physical layer performance is estimated using fully simulated LoRa receivers, described in detail in [25]. As a result, the impact of LoRa implementation details, e.g., specific parameters that can be enabled or disabled in the Semtech SX1276 LoRa transceiver used in [21,24], is ignored. Doppler shifts are considered as static and dynamic contributions in Section 3.1 and Section 3.2, respectively.

### 3.1. Static Doppler Shift

A static Doppler shift occurs when the transmitter and receiver are moving at a constant relative velocity with respect to one another. When the frequency swing *B* is assumed to be much smaller than the RF carrier frequency *F*, static Doppler shifts can be simulated by adding a frequency offset Δf to the $F_{a}\left[n,a\right]$ term in (2). Assuming a worst-case angle of incidence, the offset Δf can be calculated based on the value for *F* and the relative velocity Δv of the receiver w.r.t. the transmitter as

(7) $$\Delta f=\frac{\Delta v}{c}\;\cdot \;F,$$

where *c* denotes the speed of light.

**Figure 2:**
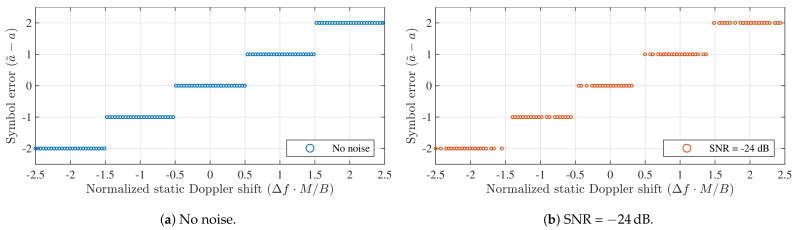
Symbol errors observed when detecting a synthesized LoRa symbol subject to different amounts of static Doppler shift. When no dot is present, the symbol error is outside [−2,2]. (SF = 12, *B* = 125 kHz).

When a frequency shift occurs as a result of the Doppler effect, the LoRa receiver’s frequency settings will no longer be matched to the one of the incoming LoRa signal. As LoRa uses linear chirps, this causes symbol energy to shift to other frequency bins. If the down-chirp of the receiver is perfectly time-aligned with the LoRa symbol and the absolute value of Δf is larger than 0.5·B/M, this will cause a symbol error. Moreover, when a significant amount of noise is present, symbol errors may occur even when |Δf|<0.5·B/M. This is demonstrated in Figure 2, which shows the symbol errors recorded when adding different Doppler shifts to a synthesized LoRa symbol (SF = 12, *B* = 125 kHz), transmitted over a noise-free channel and an additive white Gaussian noise (AWGN) channel with very adverse noise conditions (SNR = −24 dB ). As the same exercise can be performed for other spreading factors, the average frequency offsets are normalized w.r.t. *M* and *B* through Δf·M/B. However, note that the SNR values at which this performance is observed will vary when changing either of these parameters. For each SF decrement by one, or each frequency swing multiplication by two, this approximately corresponds to a 3 dB SNR performance penalty [11,25]. To provide some reference with regard to the normalized Doppler shift, Table 1 shows the denormalized frequency errors for each SF value, assuming a fixed frequency swing of 125 kHz, which is default for LoRa modulation. Table 1 also shows the corresponding relative velocities that would cause this Doppler shift for an operating frequency of *F* = 868 MHz. The values in Table 1 confirm that, for the LoRa symbol detector, the effects of Doppler shift are significantly worse for higher spreading factors. This was first shown in [21], where the coherence time of a channel impacted by Doppler shifts is expressed as a function of the relative velocity between both communicating nodes. These coherence times are compared to the duration of LoRa symbols at each spreading factor. For higher SF values, these symbol durations are significantly larger.

**Figure 3:**
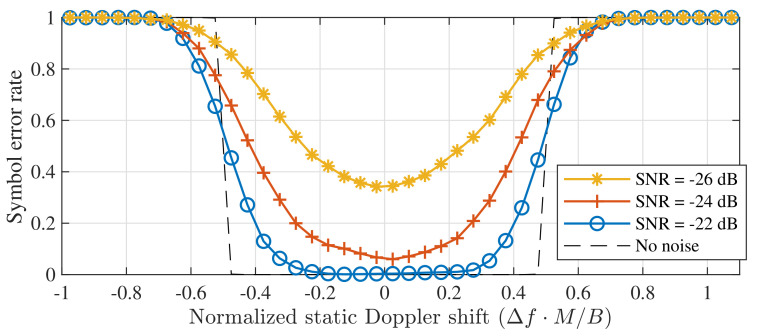
Symbol detection performance for static Doppler shifts with a perfectly time-aligned down-chirp. (SF = 12, *B* = 125 kHz).

The range of normalized Doppler shifts that remains error-free in the absence of noise in Figure 2 is explored in more detail in Figure 3. This figure shows the symbol error rate (SER) as a function of the normalized Doppler shift, defined earlier as Δf·M/B. Based on Figure 2 and Figure 3, one might be tempted to conclude that static Doppler shifts have a strong impact on the entire physical layer of LoRa modulation, even for relatively low velocities, as even a slight frequency offset might easily cause symbol errors in the detector.

**Figure 4:**
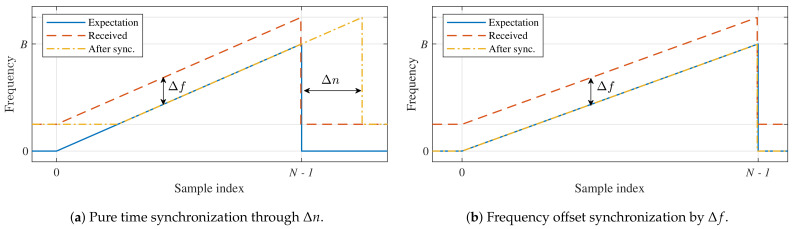
Up-chirps for time and frequency synchronization strategies that mitigate static Doppler shifts.

However, note that the discrete symbol errors shown in Figure 2 will only occur when the down-chirp is perfectly time-aligned. If the receiver performs any form of synchronization based on the timing of the pilot sequence in the packet’s preamble, this frequency mismatch will at least be reduced to the interval [−0.5,0.5]·M/B, mitigating the discrete symbol errors shown in Figure 2 if no noise is present. In practice, this very rudimentary form of synchronization introduces a relative time shift Δn/N, equal to Δf/B, as illustrated in Figure 4a. Unfortunately, countering the frequency shift solely with a time shift results in the loss of some symbol energy as the received chirp is not aligned with the frequency range (0,B) expected by the receiver. Moreover, the detection is performed in the presence of a timing error Δn, causing some intersymbol interference. Therefore, frequency errors larger than about B/2 cannot be handled when using this synchronization strategy.

**Figure 5:**
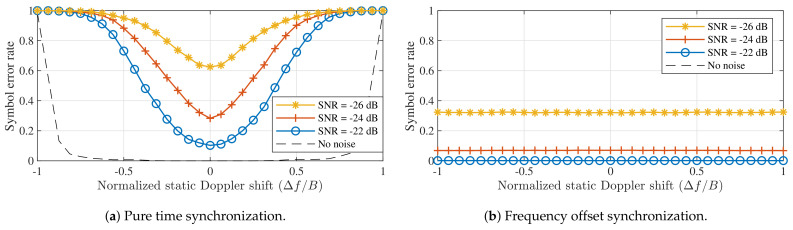
LoRa symbol detection performance when using the different synchronization strategies illustrated in Figure 4. Both strategies are presented with the value for the frequency shift applied to the synthesized symbol. Δ*n* is calculated based on this metric. (SF = 12, *B* = 125 kHz).

Alternatively, the frequency shift can be corrected by the receiver, based on the frequency contents of the preamble, as is introduced in [25] and illustrated in Figure 4b. Naturally, as presented in [25], estimating this frequency offset does come at a slightly higher computational cost. Either way, the receiver should be able to correctly process the packet as the detected symbol values shift equally across the packet w.r.t. the pilot symbols in the preamble. Yet, if no frequency correction is performed, the error performance is improved by performing time synchronization. This is confirmed in Figure 5, which shows the LoRa symbol detection performance for both strategies at different noise levels and SF = 12. Both these strategies are presented with the value for the frequency shift that was applied to the synthesized symbol. Therefore, in the time synchronization case, Δn is calculated based on this known value. Static Doppler shifts are again normalized. However, as it is no longer appropriate to normalize the frequency offset w.r.t. B/M, it is now expressed as a fraction of *B*. All simulations assume *B* = 125 kHz.

A few interesting points are demonstrated in Figure 5. First, it is shown that symbol detection performance is significantly better when applying an appropriate frequency offset instead of purely relying on time synchronization. In fact, in the latter case, symbol errors are observed for all non-zero static Doppler shifts when no noise is present. However, note that applying an appropriate coding strategy is expected to relieve part of this problem. When applying a correct receiver frequency offset, the same symbol error rate is observed for all applied Doppler shifts.

**Figure 6:**
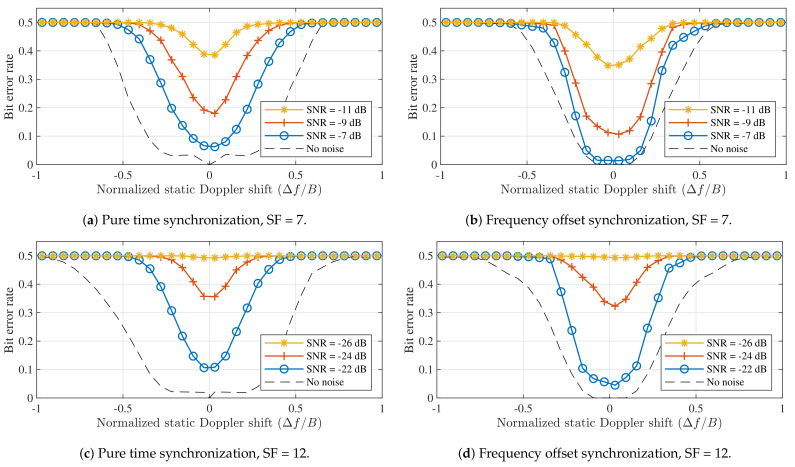
Bit error performance when using the different synchronization strategies illustrated in Figure 4 for different spreading factors. Based on synthesized LoRa packets with 100 data symbols, received by simulated LoRa receivers. (*B* = 125 kHz).

When considering the performance of LoRa modulation under static Doppler shifts, a distinction is made between the symbol error performance of the detector presented in Figure 5, which assumes perfect signal detection and synchronization performance to isolate the impact of the Doppler effect on the symbol detector, and the bit error rate (BER) performance of a simulated receiver, which performs packet detection and synchronization based on the received signal, as presented in [25]. Using simulated receivers allows us to more accurately assess the performance of actual LoRa hardware. Whereas [25] also presents advanced methods for improving the synchronization, beyond frequency offset correction, these are omitted here. The bit error rates observed for both synchronization strategies presented earlier are presented for SF = 7 and SF = 12 in Figure 6.

Figure 5 illustrated that applying frequency offset synchronization results in better SER performance than employing pure time synchronization. The disparity in performance between both strategies is also apparent in the results for the simulated receivers. In Figure 6a,c, bit errors are again observed for all non-zero Doppler shifts, even when no noise is present. In contrast, the BER is very low for normalized Doppler shifts in the range [−0.1,0.1] presented in Figure 6b,d. The limits of this range correspond to relative velocities around 4320 m/s. Therefore, static Doppler immunity is observed to be very high. Additionally, when no noise is present, Doppler immunity is slightly better for SF = 12 than for SF = 7. At very low SNRs, the comparison between spreading factors is less straightforward, mainly due to the differences in SNR performance.

In general, it can be concluded that, when the right synchronization and/or coding strategy is applied, LoRa modulation is practically immune to purely static Doppler shifts as long as these do not exceed about 10% of *B*. For body-centric LoRa networks, this means that a constant Doppler shift is expected to have very little influence on link performance for the overwhelming majority of applications due to the sheer magnitude of the relative velocities presented above. However, these results could be relevant for satellite-to-body communication or other space-related applications, where relative velocities are much higher [29]. Yet, in space applications, relative velocities are also known to a certain degree. Consequently, the operating frequencies of LoRa receivers could be adapted to correct for static Doppler shifts.

### 3.2. Dynamic Doppler Shift

As the relative velocity between two communicating nodes is rarely constant in body-centric networks, dynamic Doppler effects should also be considered. Considering a constant relative acceleration Δa of the receiver w.r.t. the transmitter during a LoRa packet, a constant Doppler rate Δα (in Hz/s) is introduced, which manifests itself as an offset Δα to be added to the slope α of the chirps described by (2). Once again assuming a worst-case angle of incidence, the Doppler rate is given by

(8) $$\Delta \alpha =\frac{\Delta a}{c}\;\cdot \;F,$$

where *F* again indicates the RF frequency of the chirp.

**Figure 7:**
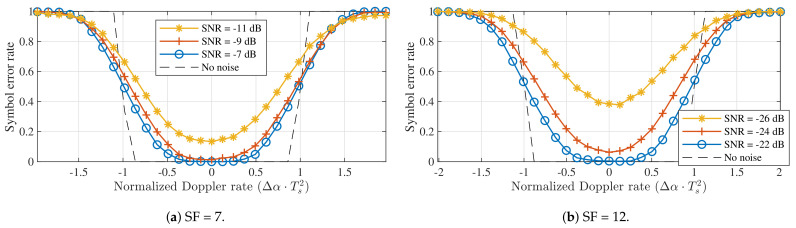
Symbol detection performance for constant Doppler rates when the down-chirp is perfectly time-aligned. (*B* = 125 kHz).

For a single LoRa symbol transmitted and received in realistic acceleration conditions, this Doppler rate is expected to have almost no influence on the performance of the symbol detector. After all, using this kind of slope offset is equivalent to communicating with a receiver utilizing a slightly different chirp rate. The symbol error rates observed for SF = 7 and SF = 12 are shown in Figure 7. These were acquired by synthesizing LoRa packets with a slope α+Δα and applying these packets to a symbol detector with a perfectly time-aligned down-chirp and no chirp rate offset. In this figure, the Doppler rate is normalized through $\Delta \alpha \;\cdot \;T_{s}^{2}$. Figure 7 confirms that the symbol detector is very robust against constant Doppler rates. In fact, when no noise is considered, errors are only observed for normalized Dopper rates outside [−1,1]. These rates correspond to acceleration values of 285 km/s${}^{2}$ and 285 m/s${}^{2}$. While the Doppler rate immunity for SF = 12 is three orders of magnitude worse than the one for SF = 7, both of these acceleration values are quite unrealistic in practice.

**Figure 8:**
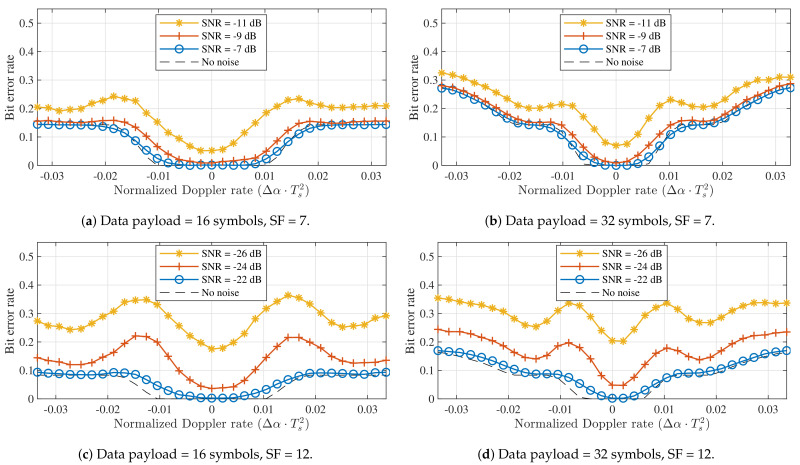
Bit error performance impact of constant Doppler rates for different data payload sizes and spreading factors (*B* = 125 kHz).

However, despite the minimal impact on symbol detection performance, it can be expected that the introduction of Δα will have a much larger effect at the packet level. As the frequency offset between the received packet and the one expected or estimated by the receiver changes over time, the impact of a constant Doppler rate will depend on the duration of the packet, which is determined by the spreading factor, the frequency swing, and the length of the data payload. As shown in Section 3.1, the static Doppler shift is mostly eliminated through packet synchronization, which is based on the contents of the preamble. Consequently, when a constant acceleration is assumed, the resulting Doppler rate will have a larger impact on symbols at the end of the data payload, while those directly following the preamble may be relatively unimpacted. To quantify this, the impact of the constant Doppler rate caused by a constant acceleration on the BER is determined for packets with different packet lengths (in number of data payload symbols). LoRa packets were synthesized with a modified chirp rate α+Δα. The receiver decoded these packets using perfectly aligned down-chirps with chirp rate α, after applying a constant frequency correction to the packet, equal to the average of the true frequency mismatch observed in the preamble of the packet. The results of these simulations are shown for SF = 7 and SF = 12 in Figure 8.

When no noise is considered, a range of acceleration values that do not lead to bit errors can be observed for each subfigure of Figure 8. For both spreading factors, the normalized Doppler rates that lead to points inside this error-free range are given by [−0.01,0.01] and [−0.006,0.006] for 16 and 32 symbol payloads, respectively. We denote the denormalized absolute values for the limits of these ranges as $\Delta \alpha_{\max}$. Reference values for $\Delta \alpha_{\max}$ and the corresponding relative acceleration values Δa are shown for all spreading factors in Table 2. These were determined by repeating the simulations presented in Figure 8 for each SF value.

**Figure 9:**
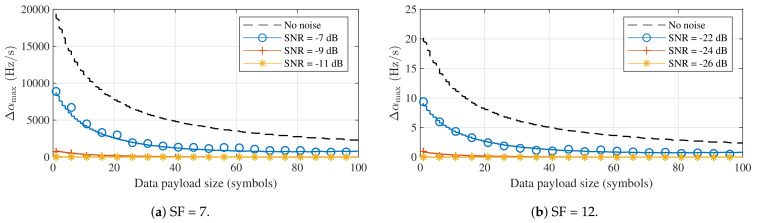
Lowest constant Doppler rate for which bit errors were observed (Δα_max_) for different data payload sizes. (*B* = 125 kHz).

Table 2 clearly shows that constant Doppler rates have a much larger impact on the BER when LoRa packets are considered. For higher spreading factors (11 and 12), errors are observed as a result of relative acceleration values that are relatively high, but not altogether unrealistic in certain body-centric wireless applications. For SF = 7, the relative accelerations that lead to bit errors are much higher, even for a hypothetical satellite-to-body communication system. The relation between the size of the data payload and the denormalized limit of the error-free range $\Delta \alpha_{\max}$ is shown for both spreading factors under study in Figure 9, where the same time synchronization and frequency correction as in Figure 8 are assumed.

**Figure 10:**
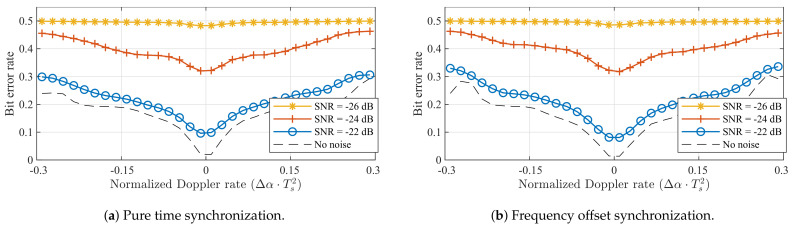
BER performance for different dynamic Doppler shifts, using the different synchronization strategies illustrated in Figure 4 (SF = 12, *B* = 125 kHz).

Finally, the impact of constant Doppler rates on LoRa receivers can be illustrated by also considering the simulated detectors presented earlier. This is realized by simulating the transmission and reception of LoRa packets (SF = 12, *B* = 125 kHz) synthesized with a certain chirp rate offset Δα. The synthesized 32-symbol payload of these packets contains a random data sequence, to which the detected symbol sequence is compared. The BER that results from this operation, shown in Figure 10, demonstrates that the full detection algorithm is similarly impacted by constant Doppler rates as the symbol detector (see Figure 8). Therefore, packet detection and synchronization are impacted less than or as much as symbol detection. In general, both receive strategies presented in Figure 4 show very similar performance. This is fully in line with expectations, as a constant Doppler rate cannot be mitigated by the type of synchronization applied in these simulated receivers. A transmit strategy that would enable LoRa receivers to alleviate constant Doppler rates caused by a constant relative acceleration could solve this by repeating a number of known pilot symbols after the data payload, effectively adding an end-of-frame delimiter. Based on the frequency offset observed for these symbols, the estimated frequency offset could be adapted dynamically to mitigate the chirp rate mismatch.

In summary, constant Doppler rates may have a larger impact on the performance of LoRa receivers than purely static Doppler shifts. In body-centric wireless applications, packet loss may occur when higher spreading factors (11 or 12) are used. For these SF values, a significant amount of bit errors are to be expected when using large packets in an environment where acceleration values are high. This occurs, for example, when using LoRa to monitor athletes in professional sports applications (skiing, cycling, skydiving, etc.) or when tracking wild animals.

## 4. Discussion

In this section, the results presented in Section 3 are compared to those of previous studies. Literature stating that LoRa modulation is very resistant to Doppler shifts possibly draws this conclusion based on indicators related to the excellent static Doppler immunity demonstrated in Section 3.1 of this paper. In experiments that led to the conclusion that LoRa is a lot more vulnerable to the Doppler effect, a multitude of factors may have impacted link performance. First of all, in addition to the relative acceleration between the communicating nodes (see Section 3), rapid changes in antenna orientation will definitely impact the link budget available for communication. Furthermore, the type of antenna that is used may also have an impact on the severity of Doppler effects, as the directivity impacts the SNR of the incoming signal. For body-centric applications, this is especially relevant as there are a lot of constraints on antenna design, the most obvious of which is its size. As already mentioned in [24], fading caused by multipath effects may also play a significant role. For example, in [21], the measurements gathered on the lathe must have been greatly impacted by multipath propagation effects, as the transmitter was placed on a radio mast outdoors. This interpretation also agrees with most other research results presented in literature. The experiments presented in [23] were indeed performed in a relatively open environment, employing a base station placed on a high office building. These factors might have alleviated multipath effects to a certain degree. In [30], results indicated that packets were lost in a LoRa body-to-body range test when the test persons were moving towards each other, while several packets were received at considerable distances while the test persons were standing still. This range test was also performed in a relatively open environment, however, both test persons were walking on the same ground level. Therefore, when more distance separated the test persons, more scatterers may have had a larger impact on the link as the direct link path got weaker. When considering multipath propagation contributions, it is again important to address the fact that also the reception and transmission of certain multipath components may be influenced by the radiation characteristics of the antennas under consideration. For example, different antenna topologies exist that actively resist multipath effects [31,32].

## 5. Conclusions

An assessment of static and dynamic Doppler effects on LoRa symbol detection performance was presented based on experiments with a simulated LoRa symbol detector that can be implemented on SDR. Additionally, two types of LoRa receivers were simulated, comparing two different synchronization strategies. For static Doppler effects, it has been shown that, while the symbol detector itself is highly vulnerable to frequency shifts, Doppler effects are largely mitigated by either of these synchronization strategies. For simulated receivers, immunity with respect to constant frequency shifts up to 10% of the frequency swing of the LoRa signal is demonstrated. As expected, the best performance is observed for a receiver that applies frequency correction. Dynamic Doppler shifts, modeled as constant Doppler rates, mainly impact LoRa communication on the packet level, as opposed to the symbol detection level, which is highly robust against constant Doppler rates. As expected, packets with a larger payload are impacted proportional to their length. When considering the impact on the performance of the symbol detector, a range of Doppler rates that do not lead bit errors is observed. For payload lengths of 16 and 32 symbols, respectively, this range corresponds to 1% and 0.6% of the inverse of the squared symbol duration. Both static and dynamic Doppler effects can be mitigated by either applying time and/or frequency synchronization, or by modifying the structure of LoRa packets by, e.g., including an end-of-frame delimiter that contains another sequence of pilot symbols, respectively.

When applying these results to body-centric LoRa applications, static Doppler shifts are expected to have very little impact on link performance, especially for lower spreading factors. Yet, large dynamic Doppler shifts may significantly reduce the quality of the wireless link for higher spreading factors when the SNR is very low. Overall, however, LoRa is still regarded as an excellent modulation technology for application in body-centric wireless networks, given the extremely low power use and high sensitivity of LoRa hardware.

## Figures and Tables

**Table 1:** Reference values for Δf and Δv for a normalized Doppler shift of 0.5. (*B* = 125 kHz and *F* = 868 MHz).

SF	Δf for * (Hz)	Δv (m/s)	Δv (km/h)
7	488.28	168.64	607.12
8	244.14	84.32	303.56
9	122.07	42.16	151.78
10	61.04	21.08	75.90
11	30.52	10.54	37.95
12	15.26	5.27	18.97

* Δ*f* · *M*/*B* = 0.5.

**Table 2:** Reference values for $\Delta \alpha_{\max}$ and Δa. (*B* = 125 kHz and *F* = 868 MHz).

**Data Payload = 16 Symbols**		
**SF**	**$\Delta \alpha_{\max}$ (Hz/s)**	**Δa (m/s^2^)**
7	9125	3152
8	2735	944.6
9	586.3	202.5
10	175.8	60.72
11	39.13	13.51
12	9.50	3.28
**Data Payload = 32 Symbols**		
**SF**	**$\Delta \alpha_{\max}$ (Hz/s)**	**Δa (m/s^2^)**
7	5625	1943
8	1563	539.8
9	391.3	135.1
10	97.63	33.72
11	23.05	7.96
12	5.75	1.98

## Data Availability

Not applicable.

## Abbreviations

The following abbreviations are used in this manuscript:

LPWAN	Low-power wide-area network
IoT	Internet of Things
SNR	Signal-to-noise ratio
ISM	Industrial, scientific and medical
SDR	Software-defined radio
CSS	Chirp spread spectrum
SF	Spreading factor
BW	Bandwidth
CR	Code rate
SFD	Start-of-frame delimiter
LoRaWAN	LoRa wide-area network
DFT	Discrete Fourier transform
AWGN	Additive white Gaussian Noise
SER	Symbol error rate
BER	Bit error rate
